# Overexpression of LIMK1 in hippocampal excitatory neurons improves synaptic plasticity and social recognition memory in APP/PS1 mice

**DOI:** 10.1186/s13041-021-00833-3

**Published:** 2021-07-27

**Authors:** Haiwang Zhang, Youssif Ben Zablah, An Liu, Dongju Lee, Haorui Zhang, Yanghong Meng, Changxi Zhou, Xingde Liu, Yiming Wang, Zhengping Jia

**Affiliations:** 1 Guizhou Medical University, Guiyang, 550000 Guizhou China; 2 Program in Neurosciences and Mental Health, The Hospital for Sick Children, Peter Gilgan Centre for Research and Learning, Toronto, ON M5S 1A8 Canada; 3 Department of Physiology, Temerty Faculty of Medicine, University of Toronto, Toronto, ON Canada; 4 The Key Laboratory of Developmental Genes and Human Disease, Ministry of Education, School of Life Science and Technology, Southeast University, Nanjing, China; 5 Department of Geriatrics, The Second Medical Center and National Clinical Research Center for Geriatric Diseases, Chinese PLA General Hospital, 28 Fuxing Road, Beijing, China; 6 Department of Cardiovascular Medicine, The Second Affiliated Hospital of Guizhou University of Traditional Chinese Medicine, Guiyang, 550001 Guizhou China; 7 Department of Psychiatry, Affiliated Hospital of Guizhou Medical University, Guiyang, 550004 Guizhou China; 8 Mental Health Education and Counseling Center for College Students, Guizhou Medical University, Guiyang, 550004 Guizhou China

**Keywords:** APP/PS1 transgenic mice, LTP, LIMK1, Cofilin, Learning and memory

## Abstract

Accumulating evidence indicates that the actin regulator cofilin is overactivated in Alzheimer’s Disease (AD), but whether this abnormality contributes to synaptic and cognitive impairments in AD is unclear. In addition, the brain region and cell types involved remain unknown. In this study, we specifically manipulate LIMK1, the key protein kinase that phosphorylates and inactivates cofilin, in the hippocampus of APP/PS1 transgenic mice. Using local injections of the AAV virus containing LIMK1 under the control of the CaMKIIα promoter, we show that expression of LIMK1 in hippocampal excitatory neurons increases cofilin phosphorylation (i.e., decreases cofilin activity), rescues impairments in long-term potentiation, and improves social memory in APP/PS1 mice. Our results suggest that deficits in LIMK1/cofilin signaling in the hippocampal excitatory neurons contribute to AD pathology and that manipulations of LIMK1/cofilin activity provide a potential therapeutic strategy to treat AD.

**Supplementary Information:**

The online version contains supplementary material available at 10.1186/s13041-021-00833-3.

## Introduction

Alzheimer’s Disease (AD) is the leading cause of dementia, but no effective treatment or cure is currently available. A prevailing hypothesis is that the production from sequential cleavage of the amyloid precursor protein (APP) by secretases and accumulation of amyloid peptides (Aβ) drives the progressive neuronal damage and cognitive impairment in AD [1]. Aβ molecules are released to the extracellular space and can aggregate to form soluble oligomers (AβO) or further accumulate into insoluble plaques [1–3]. How these peptides lead to neuronal degeneration and memory loss remains unclear; however, recent evidence indicates that soluble oligomers (AβO) are sufficient to cause synaptic deficits prior to the deposition of the plaques and these synaptic effects are the best correlates of cognitive impairments in AD [3–7]. It has been shown that AβO, but not monomers, induce loss of synaptic proteins and dendritic spines [3–9]. In addition, AβO can inhibit long-term potentiation (LTP), facilitate long-term depression, and impair memory without causing neuronal death [4–7, 9–15]. LTP is an extensively studied form of synaptic plasticity widely regarded as key mechanisms of memory formation [16, 17]. LTP deficits are also widely reported in animal models of AD [5–7]. The molecular mechanisms by which these synaptic processes are affected in AD are still poorly understood.

LIM domain kinase 1 (LIMK1) is a key signaling molecule critical for actin regulation in the brain. LIMK1 can be activated by various surface proteins, including glutamate receptors, and exerts its effects on actin dynamics by directly phosphorylating and inactivating the actin binding protein cofilin [18, 19]. Together with cofilin phosphatases, LIMK1 regulates the balance of phosphorylated (inactive) and dephosphorylated (active) cofilin to control actin reorganization [20, 21]. Perturbations of LIMK1 and cofilin have been shown to impair spine morphology, synaptic plasticity and memory, underscoring the crucial role of LIMK1/cofilin signaling in synaptic and brain function [22–25]. Recent studies have also shown that abnormalities in both LIMK1 and cofilin are associated with AD patients and animal models of AD [25–42]. For example, the level of phosphorylated cofilin was found to be decreased by the Aβ treatment as well as in the brain of AD mouse models [25, 28–38], suggesting that overactivation of cofilin may contribute to AD pathology. Interestingly, increased phosphorylated cofilin (i.e., decreased cofilin activity) has also been found to be associated with AD models under some circumstances [25, 29–33]. These results indicate complex effects of AD pathology on LIMK1/cofilin signaling, and therefore, whether and how LIMK1/cofilin contributes to AD pathogenesis remains unclear.

In this study, we investigated the role of hippocampal LIMK1 and cofilin using APP/PS1 transgenic mice. We used 3-month old mice because previous studies indicated that there were  little or no amyloid plaques detected at this age, which allowed us to examine the effects of LIMK1/cofilin before the plaque formation [43, 44]. We showed that these APP/PS1 transgenic mice exhibited impairments in social memory and LTP in the hippocampus. Hippocampal level of phosphorylated cofilin was also decreased. Viral expression of LIMK1, specifically in the hippocampal excitatory neurons, increased cofilin phosphorylation and LTP and improved social memory. These results established the critical roles of hippocampal LIMK1/cofilin in early synaptic and memory deficits in APP/PS1 mice and suggested that LIMK1/cofilin signaling pathway may serve as potential therapeutic target to treat AD.

## Results

### Impaired social memory in APP/PS1 mice

**Fig. 1:**
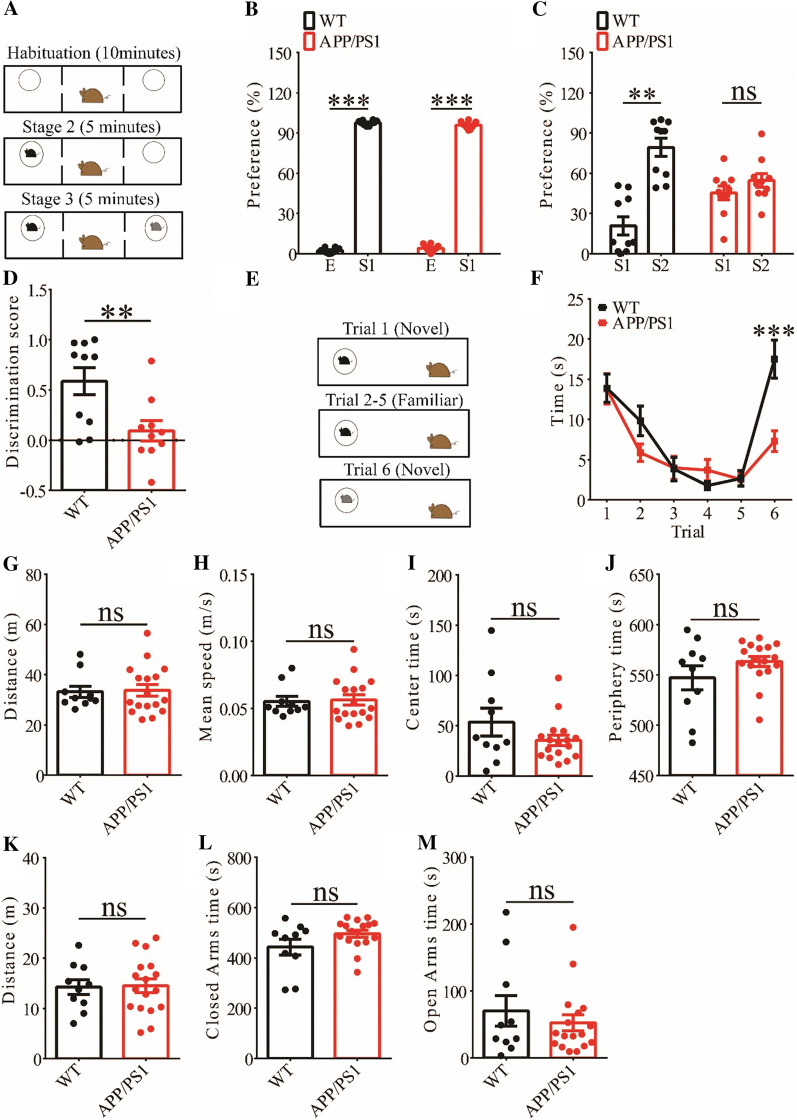
Impaired social memory in 3-month old APP/PS1 mice. **A** Schematic of the three-chamber social recognition memory test consisting of stage 1 (habituation), stage 2 (social interaction) and stage 3 (social recognition memory). **B** Normal social interaction during stage 2 in APP/PS1 mice (WT n = 10, p < 0.001; APP/PS1: n = 10, p < 0.001; two-tailed paired t-test). **C** Impaired preference for S2 over S1 during stage 3 in APP/PS1 mice (WT n = 10, p = 0.002; APP/PS1: n = 10, p = 0.386; two-tailed paired t-test). **D** Discrimination scores during stage 3 showing impaired social memory in APP/PS1 mice (p = 0.009, two-tailed t test). **E** Schematic of the five-trial social memory test. **F** Both WT and APP/PS1 mice showed memory acquisition during trials 1–5 (WT n = 10, APP/PS1 n = 17; genotype group: F_(1,9)_ = 0.002, p = 0.965; trial: F_(4,36)_ = 17.572, p < 0.001; repeated two-way ANOVA for trial 1 versus trial 5). On trial 6, APP/PS1 mice showed significantly decreased interaction time compared to WT mice during the presentation of a novel stranger mouse (p < 0.001, two-tailed t test). **G** Open field test showing travel distance in WT and APP/PS1 mice (WT n = 10, APP/PS1 n = 17, p = 0.864, two-tailed t-test). **H** Average travel speed of WT and APP/PS1 mice in open field test (WT n = 10, APP/PS1 n = 17, p = 0.851, two-tailed t-test). **I** Time spent in center arena in open field test (WT n = 10, APP/PS1 n = 17, p = 0.242, two-tailed t-test). **J** Time spent in peripheral area in open field test (WT n = 10, APP/PS1 n = 17, p = 0.235, two-tailed t-test). **K** Travel distance in elevated plus maze test (WT n = 10, APP/PS1 n = 17, p = 0.899, two-tailed t-test). **L** Time spent in closed arms in elevated plus maze test (WT n = 10, APP/PS1 n = 17, p = 0.092, two-tailed t-test). **M** Time spent in open arms in elevated plus maze test (WT n = 10, APP/PS1 n = 17, p = 0.455, two-tailed t-test). Data are presented as mean ± s.e.m. n = number of mic. **p < 0.01, ***p < 0.001, ns = not significant

We focused on social behavior and memory in 3-month old mice because this aspect is relatively less studied compared to other cognitive processes in APP/PS1 transgenic mice. We assessed social interaction and memory using the three-chamber social interaction test and the five-trial social memory test. In the three-chamber social interaction test (Fig. 1A) that consisted of three stages (stage 1: habituation; stage 2: sociability; stage 3: social memory), both APP/PS1 and wild-type (WT) littermates spent more time interacting with the stranger 1 (S1) than the empty cage, suggesting that sociability was not altered in APP/PS1 mice (Fig. 1B). However, during stage 3, while WT mice spent more time interacting with the novel stranger (S2) than S1, APP/PS1 mice spent similar amount of time interacting with S1 and S2, suggesting impaired social recognition memory (Fig. 1C, D). In the five-trial social memory test (Fig. 1E), both WT and APP/PS1 mice spent gradually less time interacting with the stranger mouse during the repeated exposures (trial 1–5), but showed increased interaction when a novel stranger mouse was introduced on trial 6 (Fig. 1F). However, APP/PS1 mice spent significantly less time interacting with the novel stranger than WT mice on trial 6 (Fig. 1F), suggesting impaired social recognition memory. These results suggest that APP/PS1 mice are impaired in social recognition memory but not sociability. We also conducted the open field test but found no significant differences between WT and APP/PS1 mice in travel distance/speed or the amount of time spent in center/periphery zone of the arena (Fig. 1G–J). Similarly, there were no differences in total travel distance and the amount of time spent in the closed or open arms during the elevated plus maze test (Fig. 1L–M). These results suggest that locomotor activity and anxiety-like behavior were not significantly altered in 3-month old APP/PS1 mice.

### Impaired LTP and reduced cofilin phosphorylation in APP/PS1 mice

**Fig. 2:**
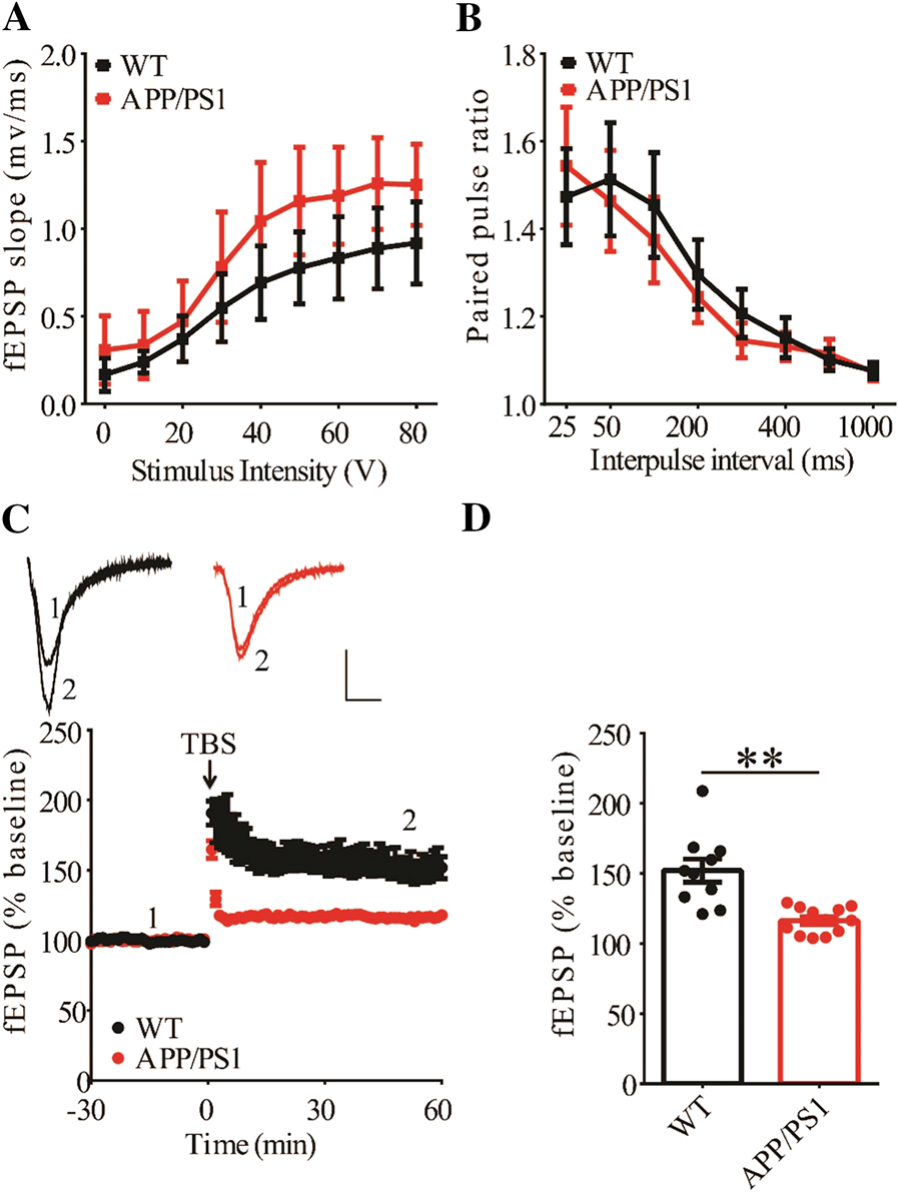
Impaired LTP in 3-month old APP/PS1 mice. **A** Input–output curves of fEPSP showing no differences between WT and APP/PS1 mice (WT n = 4 slices from 4 mice, APP/PS1 n = 6 slices from 4 mice; genotype, group: F_(1,3)_ = 1.210, p = 0.352; stimulus intensity: F_(8,24)_ = 29.777, p < 0.001; repeated two-way ANOVA). **B** Paired pulse ratio showing no differences between WT and APP/PS1 mice (WT n = 6 slices from 4 mice, APP/PS1 n = 6 slices from 4 mice; genotype: F_(1,5)_ = 0.013, p = 0.913; inter-pulse interval: F_(7,35)_ = 18.961, p < 0.001; repeated two-way ANOVA). **C** TBS induced LTP at the CA1 synapse in WT, but not in APP/PS1 mice. Scale bars: 0.2 mV/10 ms. **D** Summary graph showing no LTP in APP/PS1 compared to WT mice (WT n = 10 slices from 6 mice, APP/PS1 n = 12 slices from 7 mice, p = 0.002, two-tailed t-test). Data are presented as mean ± s.e.m. fEPSP sample traces in this and other figures were average of 6 individual responses. **p < 0.01, ns = not significant

**Fig. 3:**
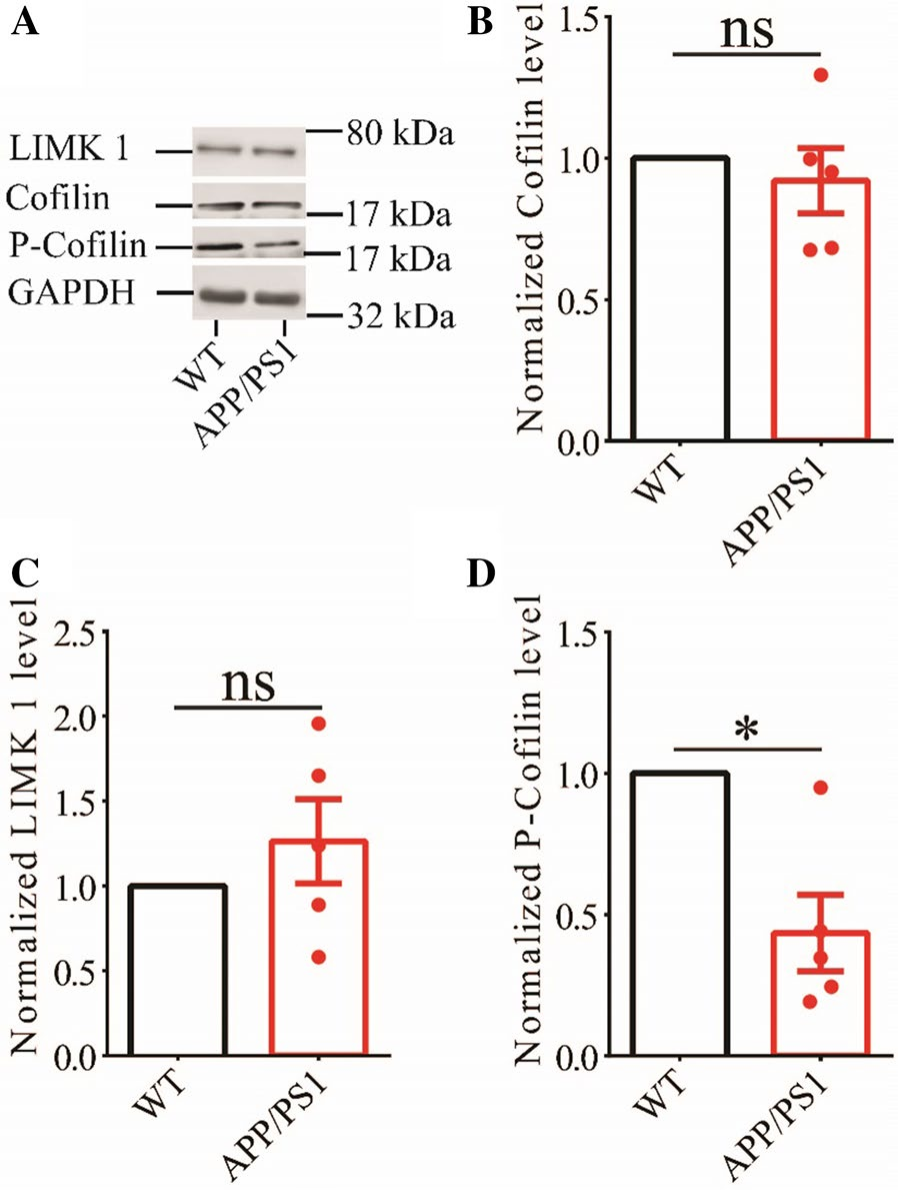
Reduced P-cofilin in the hippocampus of 3-month old APP/PS1 mice. **A** Sample Western blots of hippocampal protein lysate showing reduced protein of P-cofilin, but not total cofilin and LIMK1. **B** Summary graphs showing no significant difference in LIMK1 protein expression between WT and APP/PS1 mice (WT n = 5 independent experiments from 5 mice, APP/PS1 n = 5 independent experiments from 5 mice, p = 0.350, two-tailed t-test). **C** Summary graph showing no significant difference in total cofilin protein expression between WT and APP/PS1 mice (WT n = 5 independent experiments from 5 mice, APP/PS1 n = 5 independent experiments from 5 mice, p = 0.524, two-tailed t-test). **D** Summary graph showing significantly reduced P-cofilin protein level in APP/PS1 compared to WT mice (WT n = 5 independent experiments from 5 mice, APP/PS1 n = 5 independent experiments from 5 mice, p = 0.014, two-tailed t-test). Data are presented as mean ± s.e.m. *p < 0.05, ns = not significant

To investigate cellular mechanisms underlying memory deficits in APP/PS1 mice, we carried out electrophysiological recordings at the Schaffer collateral-commissural pathway (CA1 synapse). We first examined basal synaptic transmission using various stimulation intensities but found no differences in input/output curves of field excitatory postsynaptic potentials (fEPSPs) between WT and APP/PS1 mice (Fig. 2A). Presynaptic function as judged by paired-pulse facilitation (PPF) was also not altered in APP/PS1 mice (Fig. 2B). We then compared LTP induced by a theta burst stimulation (TBS) protocol. As shown in Fig. 2C and D, TBS-induced LTP was significantly lower in APP/PS1 compared to WT mice. These results indicate that APP/PS1 mice were impaired in synaptic plasticity at the age of 3 months. Next, we analyzed LIMK1 and cofilin expression using Western blot analysis of protein lysates prepared from the hippocampus. As shown in Fig. 3, while the total protein levels of LIMK1 and cofilin were not altered, phosphorylated (i.e., inactive) cofilin (P-Cofilin) was significantly reduced in APP/PS1 mice. Therefore, cofilin activity is abnormally upregulated in the hippocampus of APP/PS1 mice.

### Hippocampal expression of LIMK1 increases phosphorylated cofilin in APP/PS1 mice

**Fig. 4:**
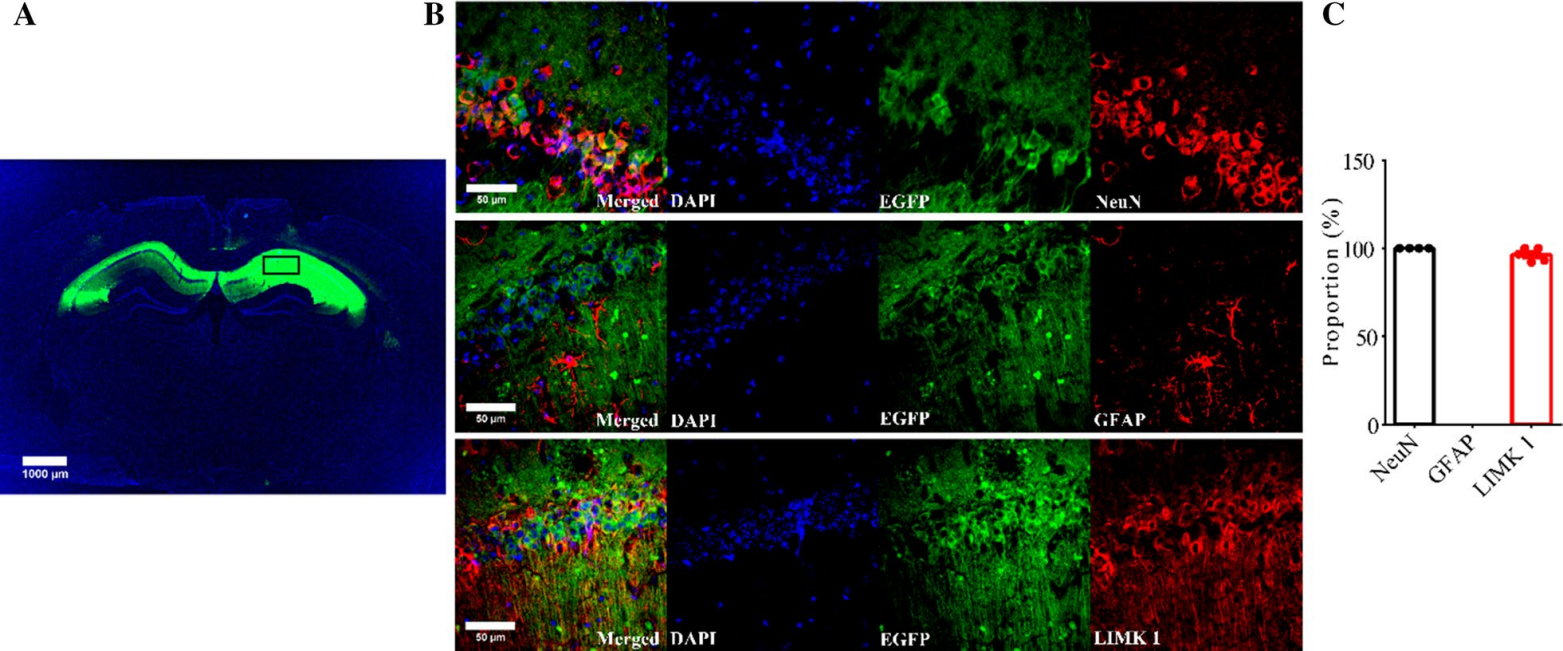
Viral expression of LIMK1-EGFP in the hippocampus. **A** Sample image showing EGFP fluorescent signals within the hippocampus of APP/PS1 mice 3 weeks after bilateral AAV virus injection (3-month old). Scale bar: 1000 μm. **B** Immunostaining images for the neuronal marker NeuN, astrocytic marker GFAP and LIMK1 showing LIMK1-EGFP signals colocalized with NeuN and LIMK1, but not with GFAP. Scale bars: 50 μm. **C** Summary graph showing proportion of LIMK1-EGFP expressing cells that also expressed NeuN, LIMK1 or GFAP (n = 5 sections from 5 mice)

**Fig. 5:**
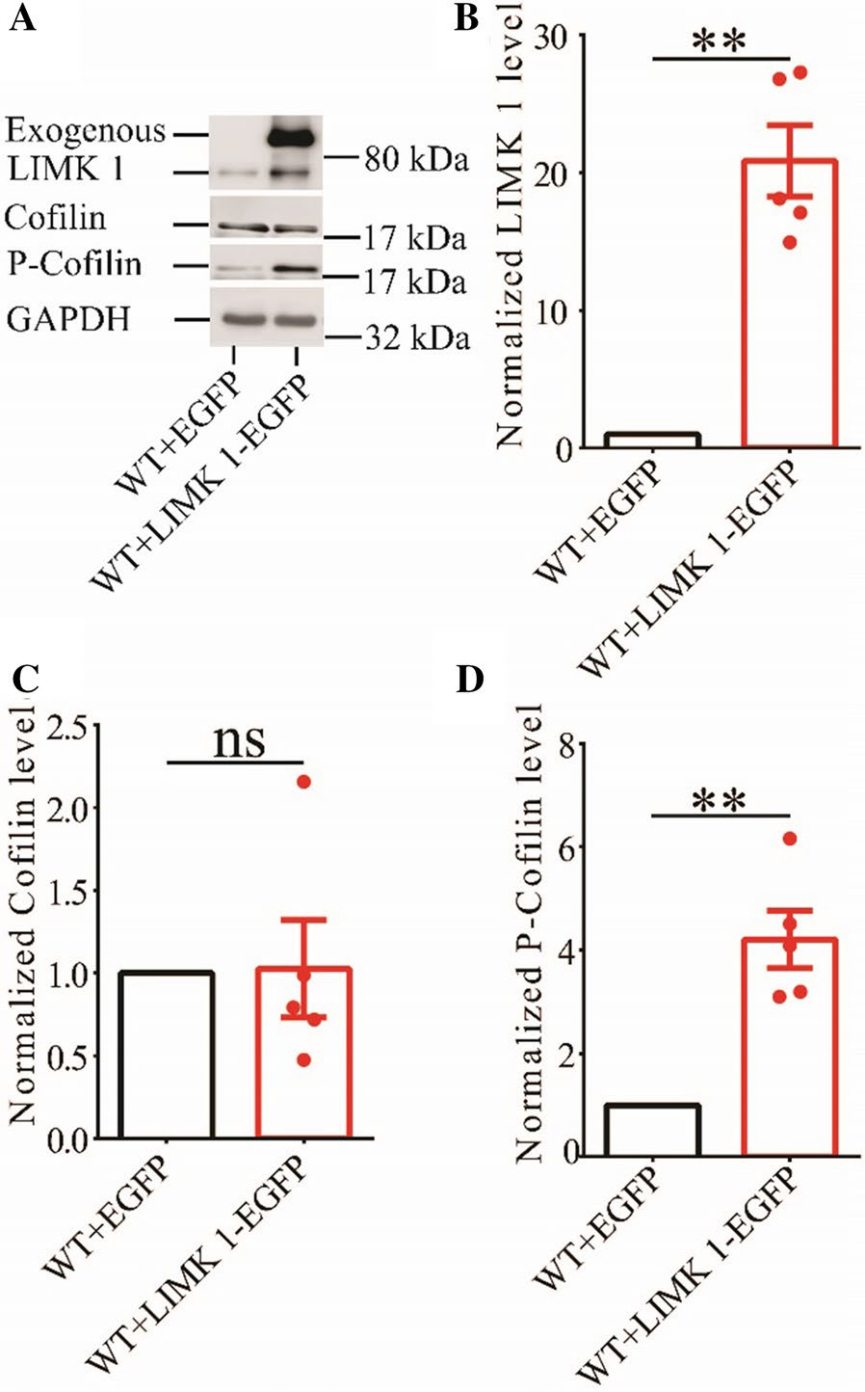
Overexpression of LIMK1-EGFP increases hippocampal P-cofilin in 3-month old WT mice. **A** Sample Western blots of hippocampal protein lysate showing protein levels of LIMK1, total cofilin and P-cofilin. **B** Summary graph showing significantly increased hippocampal LIMK1 protein level in WT mice expressing LIMK1-EGFP compared to WT mice expressing EGFP (WT + EGFP n = 5 independent experiments from 5 mice, WT + LIMK1-EGFP n = 5 independent experiments from 5 mice, p = 0.002, two-tailed t-test). **C** Summary graph showing no significant difference in total cofilin protein level between WT mice expressing EGFP and LIMK1-EGFP (WT + EGFP n = 5 independent experiments from 5 mice, WT + LIMK1-EGFP n = 5 independent experiments from 5 mice, p = 0.934, two-tailed t-test). **D** Summary graph showing significantly increased P-cofilin protein level in hippocampus in WT mice expressing LIMK1-EGFP compared to WT mice expressing EGFP (WT + EGFP n = 5 independent experiments from 5 mice, WT + LIMK1-EGFP n = 5 independent experiments, p = 0.004, two-tailed t-test). Data are presented as mean ± s.e.m. **p < 0.01, ns = not significant

**Fig. 6:**
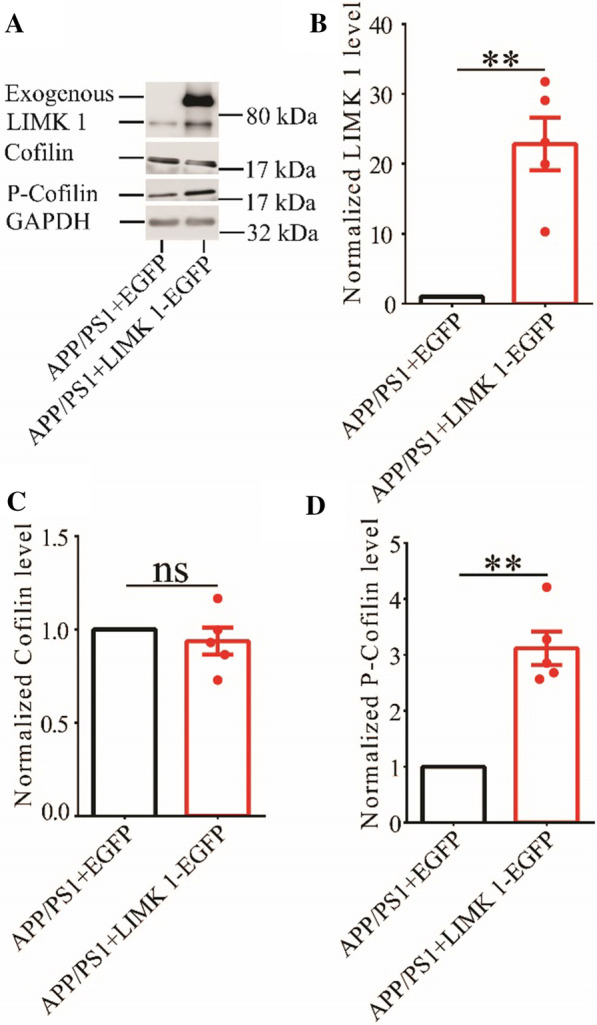
Overexpression of LIMK1-EGFP increases hippocampal P-cofilin in 3-month old APP/PS1 mice. **A** Sample Western blots of hippocampal protein lysate showing protein levels of LIMK1, total cofilin and P-cofilin in APP/PS1 mice expressing LIMK1-EGFP or EGFP. **B** Summary graph showing significantly increased hippocampal LIMK1 protein level in APP/PS1 mice expressing LIMK1-EGFP compared to WT mice expressing EGFP (APP/PS1 + EGFP n = 5 independent experiments from 5 mice, APP/PS1 + LIMK1-EGFP n = 5 independent experiments from 5 mice, p = 0.004, two-tailed t-test). **C** Summary graph showing no significant difference in total cofilin protein level between APP/PS1 mice expressing EGFP and LIMK1-EGFP (APP/PS1 + EGFP n = 5 independent experiments from 5 mice, APP/PS1 + LIMK1-EGFP n = 5 independent experiments from 5 mice, p = 0.442, two-tailed t-test). **D** Summary graph showing significantly increased P-cofilin protein level in hippocampus in APP/PS1 mice expressing LIMK1-EGFP compared to WT mice expressing EGFP (APP/PS1 + EGFP n = 5 independent experiments from 5 mice, APP/PS1 + LIMK1-EGFP n = 5 independent experiments, p = 0.002, two-tailed t-test). Data are presented as mean ± s.e.m. **p < 0.01, n = not significant

To investigate whether increased cofilin activity in the hippocampus is responsible for the synaptic and cognitive deficits in APP/PS1 mice, we employed local injections of AAV virus, which expressed LIMK1-EGFP (LIMK1 fused to EGFP) or control EGFP under the control of the excitatory neuronal promoter CaMKIIα, bilaterally into the hippocampus. We reasoned that overexpression of LIMK1-EGFP would increase cofilin phosphorylation and therefore normalize cofilin activity. As shown in Fig. 4, LIMK1-EGFP expression was restricted to the hippocampus (Fig. 4A). EGFP signals and colocalized with the neuronal marker, NeuN, as well as LIMK1, but not with the astrocytic marker, GFAP (Fig. 4B and C). To confirm the expression of LIMK1, we isolated the hippocampus and performed Western blot analysis. As shown in Figs. 5 and 6, the level of P-Cofilin was significantly increased in both WT (Fig. 5) and APP/PS1 (Fig. 6) mice expressing LIMK1-EGFP compared to EGFP control virus. Total protein level of cofilin was comparable between LIMK1-EGFP and EGFP expressing mice for both WT and APP/PS1 mice. Thus, viral expression of LIMK1 decreases cofilin activity in the hippocampus via increasing cofilin phosphorylation.

### Hippocampal expression of LIMK1 improves LTP in APP/PS1 mice

**Fig. 7:**
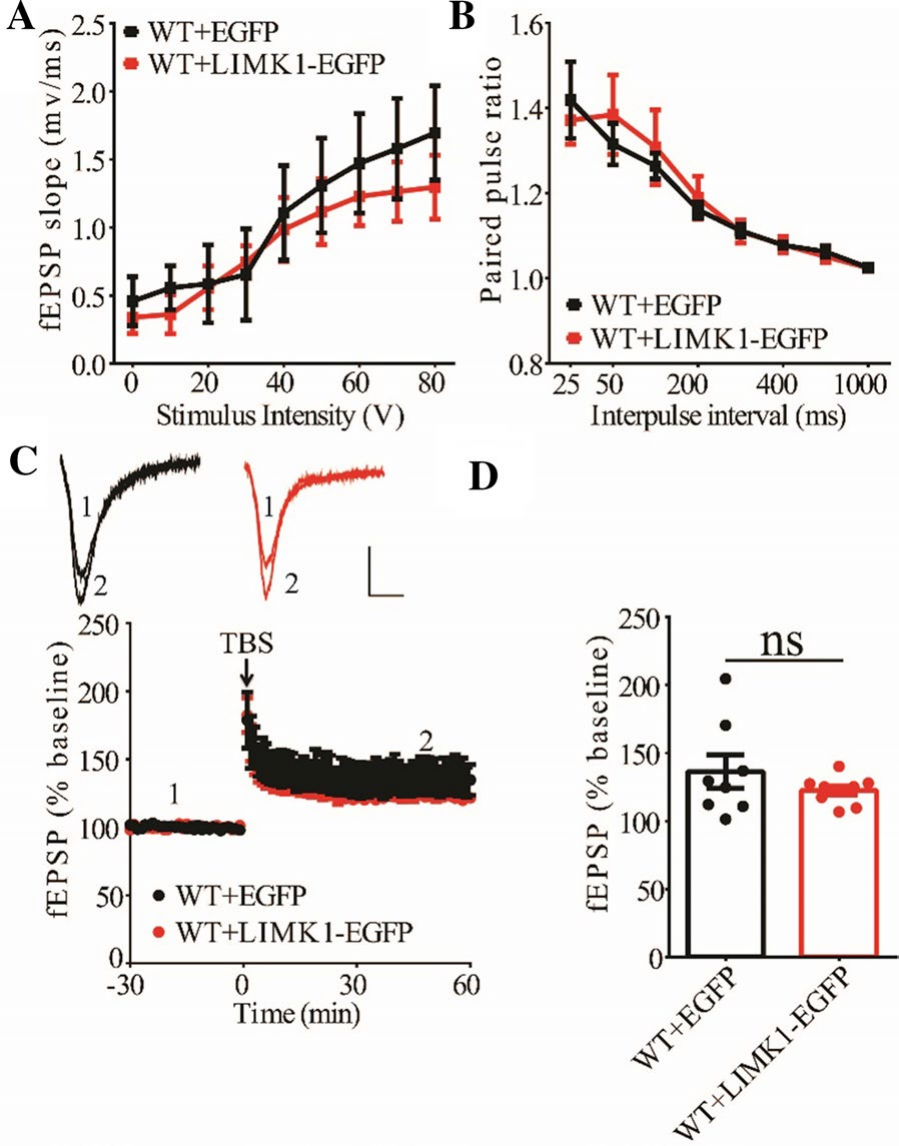
Overexpression of LIMK1-EGFP in the hippocampus has no effect on LTP in 3-month old WT mice. **A** Input–output curves of fEPSP showing no difference between WT mice expressing EGFP and LIMK1-EGFP (WT + EGFP n = 5 slices from 5 mice, WT + LIMK1-EGFP n = 6 slices from 6 mice; genotype: F_(1,3)_ = 4.626, p = 0.121; stimulus intensity: F_(8,24)_ = 24.095, p < 0.001; repeated two-way ANOVA). **B** Paired pulse ratio showing no differences between WT mice expressing EGFP and LIMK1-EGFP (WT + EGFP n = 6 slices from 5 mice, WT + LIMK1-EGFP n = 6 slices from 6 mice; genotype: F_(1,4)_ = 0.292, p = 0.617; inter-pulse interval: F_(7,28)_ = 14.844, p < 0.001; repeated two-way ANOVA). Scale bars: 0.2 mV/10 ms. **C** TBS induced comparable LTP at the CA1 synapse in WT mice expressing EGFP or LIMK1-EGFP. **D** Summary graph showing no significant difference in LTP of last 10 min between WT expressing EGFP and LIMK1-EGFP (WT + EGFP n = 8 slices from 5 mice, WT + LIMK1-EGFP n = 9 slices from 5 mice, p = 0.308, two-tailed t-test). Data are presented as mean ± s.e.m. ns = not significant

**Fig. 8:**
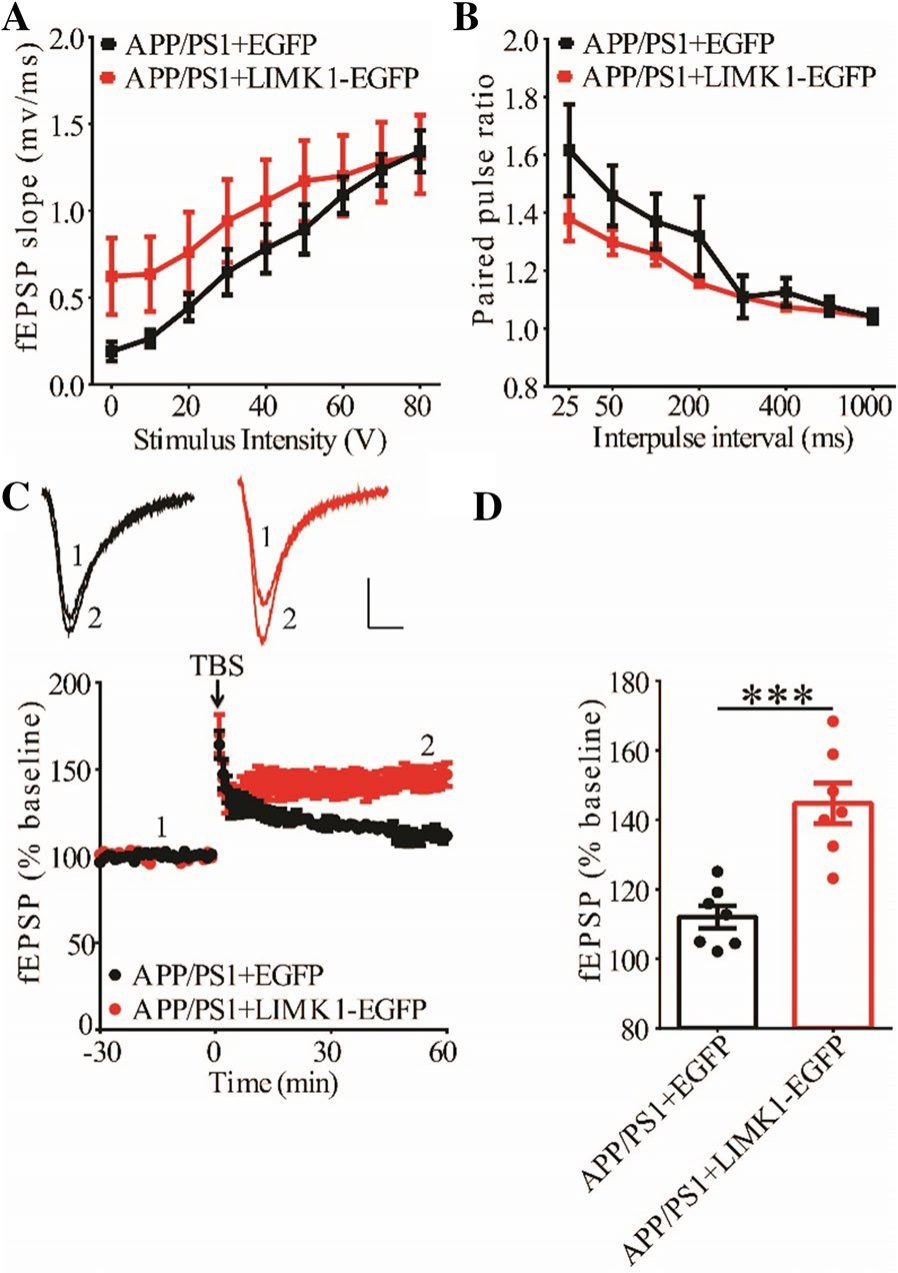
Overexpression of LIMK1-EGFP in the hippocampus improves LTP in 3-month old APP/PS1 mice. **A** Input–output curves of fEPSP showing no differences between APP/PS1 mice expressing EGFP and LIMK1-EGFP (APP/PS1 + EGFP n = 5 slices from 5 mice, APP/PS1 + LIMK1-EGFP n = 6 slices from 6 mice; genotype: F_(1,4)_ = 0.751, p = 0.435; stimulus intensity: F_(8,32)_ = 55.649, p < 0.001; repeated two-way ANOVA). **B** Paired-pulse ratio analysis showing no difference between APP/PS1 mice expressing EGFP and LIMK1-EGFP (APP/PS1 + EGFP n = 6 slices from 5 mice, APP/PS1 + LIMK1-EGFP n = 6 slices from 6 mice; genotype: F_(1,6)_ = 1.514, p = 0.265; inter-pulse interval: F_(7,42)_ = 21.588, p < 0.001; repeated two-way ANOVA). Scale bars: 0.2 mV/10 ms. **C** TBS induced higher LTP in APP/PS1 mice expressing LIMK1-EGFP compared to APP/PS1 mice expressing EGFP. **D** Summary graph showing significantly enhanced LTP of last 10 min in APP/PS1 mice expressing LIMK1-EGFP compared to APP/PS1 mice expressing EGFP (APP/PS1 + EGFP n = 7 slices from 5 mice; APP/PS1 + LIMK1-EGFP n = 7 slices from 5 mice; p < 0.001, two-tailed t-test). Data are presented as mean ± s.e.m. ***p < 0.001, ns = not significant

To investigate the effects of decreased cofilin activity caused by overexpressing LIMK1, we first examined synaptic function at CA1 synapses in both WT and APP/PS1 mice. As shown in Fig. 7, expression of LIMK1-EGFP had no effects on basal synaptic transmission, PPF and TBS-LTP in WT mice. In contrast, expression of LIMK1-EGFP significantly enhanced TBS-LTP without affecting basal synaptic strength or PPF (Fig. 8) in APP/PS1 mice. These results suggest that decreasing cofilin activity was sufficient to rescue the TBS-LTP impairment in APP/PS1 mice. It is possible that in WT mice, endogenous cofilin activity is already low that further reduction does not affect LTP.

### Hippocampal expression of LIMK1 improves social memory in APP/PS1 mice

**Fig. 9:**
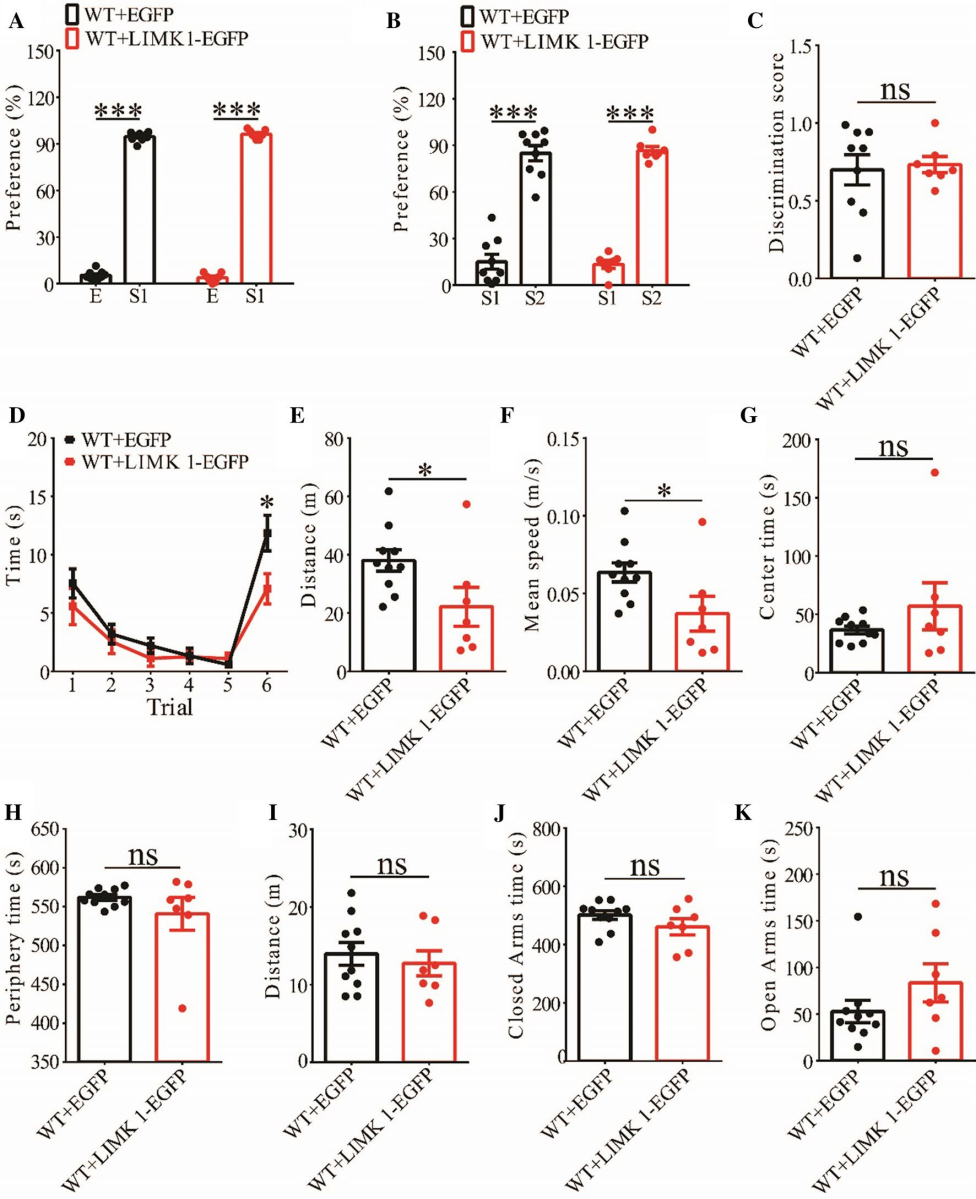
Effect of LIMK1-EGFP expression in 3-month old WT mice. **A** Normal social interaction during stage 2 of the three-chamber social test in WT mice expressing EGFP or LIMK1-EGFP (WT + EGFP n = 9, p < 0.001; WT + LIMK1-EGFP n = 7, p < 0.001; two-tailed paired t-test). **B** Preference for S2 over S1 during stage 3 of the three-chamber social test in WT mice expressing EGFP or LIMK1-EGFP (WT + EGFP n = 9, p < 0.001; WT + LIMK1-EGFP n = 7, p < 0.001; two-tailed paired t test). **C** Discrimination scores during stage 3 of three-chamber social test showing no difference in social memory between WT mice expressing EGFP and LIMK1-EGFP (p = 0.783, two-tailed t-test). **D** Both EGFP and LIMK1-EGFP expressing WT mice showed memory acquisition during trials 1–5 of the five-trial social memory assay (WT + EGFP n = 10, WT + LIMK1-EGFP n = 7; genotype: F_(1,6)_ = 7.086, p = 0.037; trial: F_(4,24)_ = 9.180, p < 0.001; repeated two-way ANOVA for trial 1–5). On trial 6, WT + LIMK1-EGFP mice showed significantly decreased interaction time compared during the presentation of a novel mouse compared to WT + EGFP mice (p = 0.041, two-tailed t-test). **E** Open field test showing travel distance in WT mice expressing EGFP or LIMK1-EGFP (WT + EGFP n = 10, WT + LIMK1-EGFP n = 7, p = 0.040, two-tailed t-test). **F** Average travel speed in pen field test (WT + EGFP n = 10, WT + LIMK1-EGFP n = 7, p = 0.040, two-tailed t-test). **G** Time spent in center arena in open field test (WT + EGFP n = 10, WT + LIMK1-EGFP n = 7, p = 0.360, two-tailed t-test). **H** Time spent in peripheral arena in open field test (WT + EGFP n = 10, WT + LIMK1-EGFP n = 7, p = 0.259, two-tailed t-test). **I** Travel distance in elevated plus maze test (WT + EGFP n = 10, WT + LIMK1-EGFP n = 7, p = 0.594, two-tailed t-test). **J** Time spent in closed arms in elevated plus maze test (WT + EGFP n = 10, WT + LIMK1-EGFP n = 7, p = 0.187, two-tailed t-test). **K** Time spent in open arms in elevated plus maze test (WT + EGFP n = 10, WT + LIMK1-EGFP n = 7, p = 0.188, two-tailed t-test). Data are presented as mean ± s.e.m. n = number of mice. *p < 0.05, ***p < 0.001, ns = not significant

**Fig. 10:**
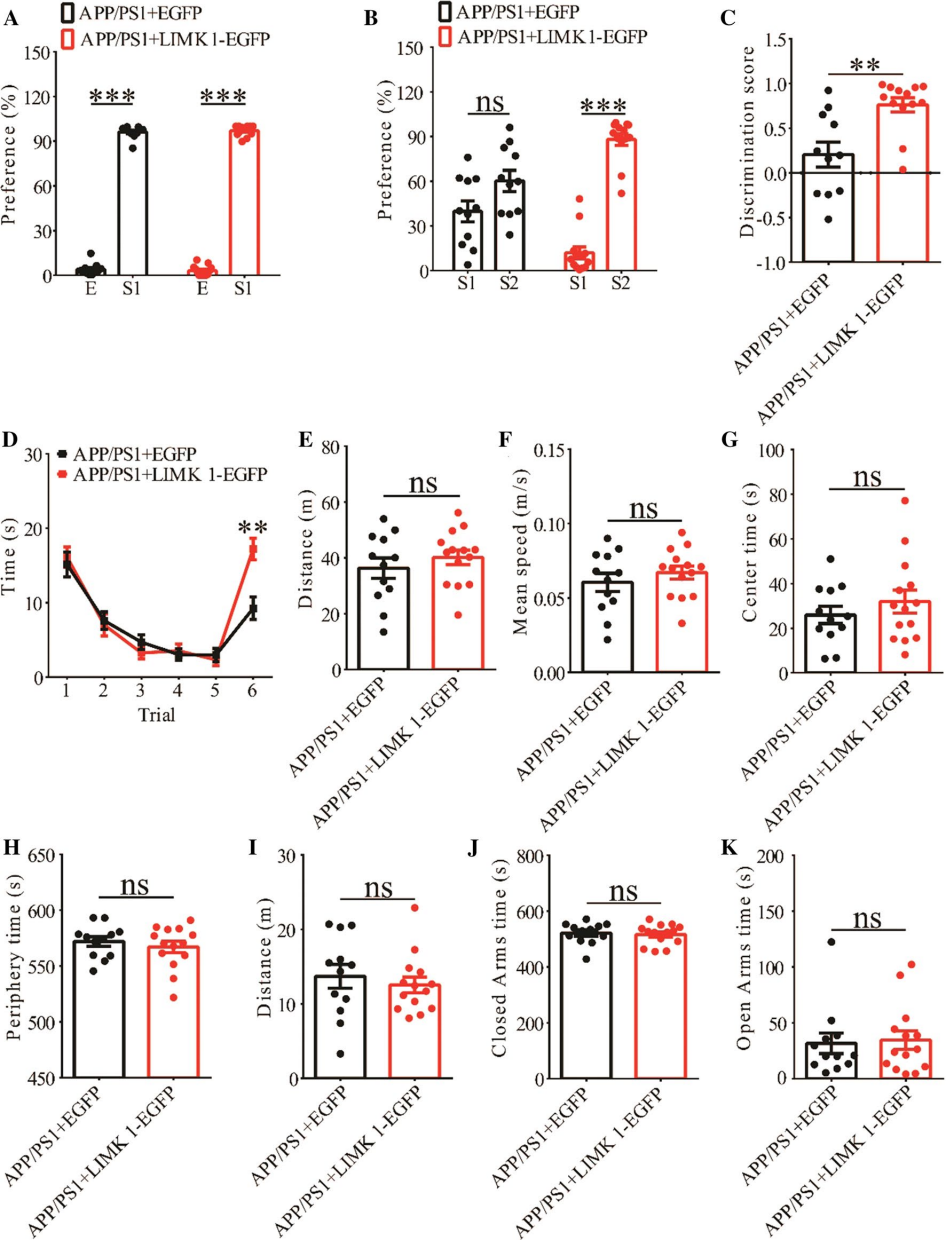
Overexpression of LIMK1-EGFP improves social memory in 3-month old APP/PS1 mice. **A** Normal social interaction during stage 2 of the three-chamber social test in APP/PS1 mice expressing EGFP or LIMK1-EGFP (APP/PS1 + EGFP n = 11, p < 0.001; APP/PS1 + LIMK1-EGFP n = 13, p < 0.001; two-tailed paired t-test). **B** Preference for S2 over S1 during stage 3 of three-chamber social test in APP/PS1 mice expressing LIMK1-EGFP but not in APP/PS1 mice expressing EGFP (APP/PS1 + EGFP n = 11, p = 0.178; APP/PS1 + LIMK1-EGFP n = 13, p < 0.001; two-tailed paired t test). **C** Discrimination scores during stage 3 of the three-chamber social test showing significantly improved social memory in APP/PS1 mice expressing LIMK1-EGFP compared to APP/PS1 mice expressing EGFP (p = 0.002, two-tailed t-test). **D** Both APP/PS1 + LIMK1-EGFP and APP/PS1 + EGFP mice showed memory acquisition during trials 1–5 of the five-trial social memory assay (APP/PS1 + EGFP n = 12, APP/PS1 + LIMK1-EGFP n = 14; genotype: F_(1,11)_ = 0.027, p = 0.873; trial: F_(4,44)_ = 59.429, p < 0.001; repeated two-way ANOVA). On trial 6, APP/PS1 + LIMK1-EGFP mice showed significantly more interaction time during the presentation of a novel stranger mouse compared to APP/PS1 + EGFP mice (p = 0.001, two-tailed t-test). **E** Open field test showing travel distance in APP/PS1 mice expressing EGFP or LIMK1-EGFP (APP/PS1 + EGFP n = 12, APP/PS1 + LIMK1-EGFP n = 14, p = 0.393, two-tailed t-test). **F** Average travel speed in pen field test (APP/PS1 + EGFP n = 12, APP/PS1 + LIMK1-EGFP n = 14, p = 0.380, two-tailed t-test). **G** Time spent in center arena in open field test (APP/PS1 + EGFP n = 12, APP/PS1 + LIMK1-EGFP n = 14, p = 0.371, two-tailed t-test). **H** Time spent in peripheral arena in open field test (APP/PS1 + EGFP n = 12, APP/PS1 + LIMK1-EGFP n = 14, p = 0.474, two-tailed t-test). **I** Travel distance in elevated plus maze test (APP/PS1 + EGFP n = 12, APP/PS1 + LIMK1-EGFP n = 14, p = 0.543, two-tailed t-test). **J** Time spent in closed arms in elevated plus maze test (APP/PS1 + EGFP n = 12, APP/PS1 + LIMK1-EGFP n = 14, p = 0.725, two-tailed t- test). **K** Time spent in open arms in elevated plus maze test (APP/PS1 + EGFP n = 12, APP/PS1 + LIMK1-EGFP n = 14, p = 0.818; two-tailed t-test). Data are presented as mean ± s.e.m. n = number of mice. **p < 0.01, ***p < 0.001, ns = not significant

To further investigate the functional consequence of overexpressing LIMK1, we examined behavioral responses in both WT and APP/PS1 mice expressing EGFP or LIMK1-EGFP. Expression of LIMK1-EGFP had no effects on social recognition memory in the three-chamber test or anxiety-like behavior in the elevated plus maze test in WT mice (Fig. 9A–C, I-K), but impaired social recognition in the five-trial test (Fig. 9D) and decreased locomotor activity in the open field test (Fig. 9E–H). These results indicate that overexpression of LIMK1 in WT mice negatively affected social behavior, suggesting that a balanced level of P-Cofilin is important. In contrast, expression of LIMK1-EGFP in APP/PS1 mice significantly improved social recognition memory in both three-chamber and five-trial repeated exposure tests (Fig. 10A–D), without affecting locomotor activity or anxiety-like behavior (Fig. 10E–K). Therefore, the elevated cofilin activity in the hippocampus is an important cause for impaired social memory in APP/PS1 mice.

## Discussion

Although changes in LIMK1/cofilin signaling in animal models of AD appear to be complex being affected by subcellular compartments and age of the mice [25, 27, 30–33], increased cofilin activity as determined by decreased P-Cofilin and the formation of aberrant cofilin-actin rods (characterized by accumulation of active cofilin) are fairly consistent observations [25, 28–40]. In addition, reducing cofilin activity by expressing constitutively inactive cofilin (S3D) [41] or manipulating cofilin regulators can rescue Aβ42-induced spine loss as well as deficits in LTP and contextual memory in APP/PS1 mice [38, 39]. Furthermore, a phosphorylated cofilin peptide that presumably prevents cofilin activation also partially improves working memory and novel object recognition in the 5XFAD AD mouse model [42]. These studies suggest that increased cofilin activity may be responsible for the cognitive and synaptic deficits associated with AD, but no direct evidence is yet available by using genetic manipulations of LIMK1/cofilin signaling. In addition, the brain regions and cell types that are involved remain unknown.

In this study, we have addressed these questions by expressing full-length LIMK1 specifically in hippocampal excitatory neurons. In order to avoid the effect of amyloid plaques, we choose to use three-month old mice because little or no plaque formations have been detected at this age [43, 44, Additional file 1: Figure S1]. First, we show that APP/PS1 mice are impaired in social recognition memory. The social memory impairments in APP/PS1 mice are evident in both the three-chamber and five-trial social interaction tasks. In both tests, sociability appears not affected. Second, we show that LTP at CA1 synapse is significantly reduced whereas basal synaptic strength and presynaptic function are intact. The impaired LTP is accompanied by increased cofilin activity without changes in total LIMK1 or cofilin in the hippocampus. These results are consistent with many previous studies [4–7] showing that synaptic and cognitive deficits occur in APP/PS1 mice even before Aβ plaque formation. Third, we show that overexpression of LIMK1 in hippocampal excitatory neurons decreases cofilin activity and improves LTP in APP/PS1 mice. Interestingly, overexpression of LIMK1 in WT hippocampus does not improve LTP although it does decrease cofilin activity. It is possible that the endogenous cofilin activity is already sufficiently low in WT mice that a further decrease does not affect LTP. However, overexpression of LIMK1 in WT hippocampus does negatively affect social memory in the five-trial test, suggesting that this effect is cofilin-independent. Finally, we show that overexpression of LIMK1 improves social memory in APP/PS1 mice. These results suggest that the abnormally high cofilin activity in 3-month APP/PS1 mice is an important contributor to the synaptic and cognitive impairments in these mice. Because the expression of LIMK1 is restricted to the hippocampal excitatory neurons, our results reveal that increased cofilin activity in hippocampal excitatory neurons may be particularly important for early synaptic and cognitive deficits in APP/PS1 transgenic mice.

Previous studies have shown that Aβ42 oligomers can activate LIMK1 and its upstream regulators (e.g., the Rho GTPases Rac1 and their associated protein kinases p21- activated kinases and Rho-kinase ROCK2) in neurons [26, 27, 31]. In addition, increased LIMK1 and ROCK2 activity has also been reported in the hAPPJ20 AD mouse model [27]. Consistent with these results, pharmacological inhibition of LIMK1 has been shown to prevent dendritic spines loss induced by Aβ42 oligomers as well as spine loss in the hAPPJ20 mouse model [27]. These results appear to be inconsistent with findings in the present study where increasing LIMK1 rescues synaptic and cognitive deficits. However, there are a number of potential reasons that could explain the discrepancy between the pharmacological inhibition and our study. First, the animal models (APP/PS1 versus hAPPJ20) used in these two studies are different and therefore the changes in LIMK1/cofilin signaling could be different. Second, the age of mice used in these studies (3-month versus 6-month old) is also different. It is known that changes in LIMK1/cofilin signaling are affected by the age of the animals [30]. Third, the present study uses molecular manipulations specifically in the hippocampal excitatory neurons rather than global inhibition of LIMK1. It is possible that changes in LIMK1/cofilin is region/cell-specific, so that global and region/cell cell-specific manipulations of LIMK1 may have different effects on synaptic and cognitive impairments in these mice. Previous studies have shown that both dorsal and ventral hippocampal regions, including dorsal CA2 and dentate gyrus (DG) as well as ventral CA1, are important for social recognition memory [45–47]. Although we injected the virus in the dorsal hippocampus, which suggests a critical role of LIMK1 in this region, it remains unknown whether dorsal CA2 or DG or both are required. It would be important to distinguish these possibilities in future studies.

In summary, we have demonstrated that cofilin activity is increased in 3-month old APP/PS1 transgenic mice and that overexpression of LIMK1, specifically in the hippocampal excitatory neurons, improves synaptic plasticity and social memory. These results suggest that changes in LIMK1/cofilin in hippocampal excitatory neurons are a key event that may underlie the neuropathology of 3-month old APP/PS1 mice before the amyloid plaque formation. It remains to be investigated whether these changes are present at other ages or in other AD models and whether manipulations of LIMK1 have a similar rescuing effect. Nevertheless, our results suggest that intervention of LIMK1/cofilin may provide a potential strategy to improve synaptic function and memory impairment associated with AD patients.

## Materials and methods

### Animals

APP/PS1 transgenic mice (#34829-JAX) were obtained from the Jackson Laboratory and were housed (2–5 mice per cage) on a 12 h/12 h light/dark cycle with food and water ad libitum. The following PCR primers were used for genotyping APP/PS1 mice: oIMR 1644: AATAGAGAACGGCAGGAGCA; oIMR 1645:GCCATGAGGGCACTAATCAT. All experimental procedures were conducted according to the guidelines of the Canadian Council on Animal Care (CCAC) and approved by the Animal Care Committee at the Hospital for Sick Children, Canada. All experiments were performed blind to the genotype of the mice. Both male and female mice were used but no differences were noted between sexes, therefore the data were pooled together for statistical analyses between genotypes.

### Surgical procedures

For virus injections, the AAV2/DJ-CaMKIIα-LIMK1-EGFP (LIMK1 fused to EGFP, 7.6 × 10^12^) and AAV2/DJ-CaMKIIα-EGFP (1.3 × 10^13^) (produced through Canadian Neurophotonics Platform, Laval University, Canada) were injected bilaterally to the dorsal hippocampus as previously described [46]. Briefly, mice were anaesthetized with isoflurane (1.5–2% in 1 L/minute oxygen) and placed onto a stereotaxic frame. Body temperature was maintained at 37 °C using a temperature controller. A midline scalp incision was made followed by craniotomies using a 0.6 mm drill bit. The viruses were injected into the dorsal hippocampus (AP: − 2.00 mm, DV: − 1.50 mm, ML: ± 1.50 mm relative to bregma; coordinates derived from Paxinos and Franklin, 2007). 1 μL of the virus was infused bilaterally at a rate of 0.5 μL/minute via an internal cannula connected by tygon tubing to a 10 μL Hamilton needle syringe. After infusion, the internal cannula was left in place for 8 min to allow for diffusion. The surgically operated mice were recovered for 4 and 6 weeks to allow for LIMK1-EGFP and EGFP expression before behaviour tests were performed. The expression pattern of LIMK1-EGFP and EGFP as well as the injection sites were confirmed by immunohistochemical staining of fixed brain sections after behaviour tests.

### Slice electrophysiology

All the electrophysiological recordings were done at the Schaffer collateral-commissural pathway (CA1 synapse) in the hippocampus as previously described [22, 48]. In brief, the mouse brains were removed and 350 µm brain slices prepared in ice-cold artificial cerebrospinal fluid (ACSF) saturated with 95% O2/5% CO2. ACSF contained (in mM): 120.0 NaCl, 3.0 KCl, 1.2 MgSO_4_, 1.0 NaH_2_PO_4_, 26.0 NaHCO_3_, 2.0 CaCl_2_, and 11.0 D-glucose. The slices were recovered at 28℃ for 15 min then for at least 2 h at room temperature before a single slice was transferred to a submersion chamber constantly perfused with 95% O2/5%CO2 saturated ACSF. Perfusion flow rate was maintained at 2 ml/min. Synaptic transmission was evoked by stimulation at 0.067 Hz and recorded with glass pipettes (3–4 MΩ) filled with ACSF. For input–output experiments, the stimulus intensity was increased gradually. Paired-pulse facilitations were obtained at inter-pulse intervals of 25 ms, 50 ms, 100 ms, 200 ms, 500 ms or 1 s, and calculated as the ratios of the second response peak values over the first response peak values. LTP was induced by three trains of theta burst stimulations (TBS, five pulses at 100 Hz every 200 ms) with an intertrain interval of 10 s. LTP was calculated and statistically evaluated by comparing the mean values of the last 10 min of the recording and the mean values of the entire baseline. All data acquisition and analysis were done using pCLAMP 10.6 (Axon Instruments, Foster City, California, USA).

### Western blot analysis

Protein lysates were prepared from hippocampal slices as previously described [48]. Briefly, hippocampus was isolated quickly on ice and dissolved in ice-cold lysis buffer containing (in mM): 20 Tris–HCl (pH 7.5), 150 NaCl, 1 EDTA, 1 EGTA, 1%Triton X-100, 1 Na3VO4, 20 NaF, and 1% protease inhibitor cocktail and phosphatase inhibitor (Roche) and kept at 4 ℃ for 40 min and debris was removed by centrifugation at 10,000*g* for 15 min. The protein samples were mixed with 25% volume of 4 × SDS loading buffer (250 mM Tris–HCl, 10% SDS, 0.5% bromophenol blue, 50% glycerol, 5% beta-mercaptoethanol, pH 7.4) and loaded and separated on a SDS-PAGE ployacrylamide gel followed by electrotransfer to a nitrocellulose filter. The filter was then blocked with 5% dry milk in TBST (20 mM Tris–HCl, 9% NaCl, 1% Tween-20, pH 7.6) and incubated overnight at 4 °C with indicated primary antibodies in TBST. Following washing and incubation with appropriate secondary antibodies, the filter was washed and developed using an enhanced chemiluminescence (Thermo-Fisher, CAT#34579) detection method. The images were analyzed using the image studio software as per manufacturer’s instruction. The amount of total protein loaded was controlled by normalizing each tested protein with anti-GAPDH immunoreactivity on the same blot. The antibodies used included: anti-Cofilin (Cell Signaling Technology, rabbit, CAT#51755), anti-LIMK1 (Cell Signaling Technology, rabbit, CAT#3842S), anti-P-Cofilin (Santa Cruz Biotechnology, rabbit, CAT#LO115), anti-GAPDH (Cell Signaling Technology, rabbit, CAT#2118S), and HRP-linked goat anti-rabbit IgG (Cell Signaling Technology, CAT#7074S).

### Immunohistochemistry

Previously described procedure was followed [46]. Briefly, mice were anesthetized by ketamine and perfused with 0.1M phosphate-buffered saline (PBS) followed by 4% paraformaldehyde (PFA in PBS). The brain was then dissected and post-fixed in 4% PFA for 24 h, and then transferred to 30% sucrose in PBS solution until it was fully saturated. The brain was then embedded in Tissue-Tek® O.C.T. compound, frozen in liquid nitrogen and sliced to 25 μm coronal or horizontal cryostat sections at − 20 °C (Leica CM1950, Concord, Ontario, Canada). The brain sections were transferred to a glass slide. Sections were washed with PBS, permeabilized by 0.3% Triton for 1 h, blocked with 5% fetal bovine serum for 1 h, and incubated with primary antibodies overnight at 4 °C, and then appropriate secondary antibodies at room temperature for 2 h. After washing, the coverslips were mounted using the ProLong Diamond Antifade mounting medium DAPI for imaging. For thioflavin-S staining, brain sections were sequentially washed in 70% and 80% ethanol for 5 min each followed by incubation in 1 thioflavin-S for 8 min at room temperature. The sections were then washed with 80%, 70% ethanol and PBS again for 5 min each. The slides were dried and cover slipped with mounting media. Images were collected on a Leica epi-fluorescence microscope and a Nikon A1R confocal microscopes and analyzed using ImageJ software (NIH, Betheseda, Maryland, USA). Primary antibodies included: anti-LIMK1 (Cell Signaling Technology, CAT#3842S), anti-NeuN (Cell Signaling Technology, CAT#12943S) and anti-GFAP (Cell Signaling Technology, CAT#3670S). Secondary antibodies included: Alexa Fluor 555 anti-rabbit IgG for anti-LIMK1 and anti-NeuN primary antibodies (Thermofisher Scientific, CAT#A31572), and Alexa Fluor 555 anti-rabbit IgG for anti-GFAP primary antibody (Thermofisher Scientific, CAT#A31570). The excitation/emission used were 402/460 (nm) for DAPI, 488/509 (nm) for GFP, and 562/580 (nm) for Alexa Fluor 555.

### Behavioral tests

All the mice were tested at the age of 3 ± 0.5 months. The mice were injected with viruses at the age of 8 weeks and behavioral tests were performed 4 weeks later. All behavioral tests were performed during the light cycle. The mice were tested in open field, elevated plus maze, three-chamber social interaction and five-trial repeated social test. At least three day intervals were given after each test. The detailed procedures of these tests were described previously [45], but briefly explained below.

The open field apparatus was a rectangular Plexiglas box (40 cm long × 40 cm wide × 35 cm high) comprising four walls and an open roof. The illumination in the room was provided by centrally placed in-ceiling dim lights. All mice were individually tested in one 5 min session. Each subject was introduced to the apparatus in the same place of the arena near the center and allowed to explore the apparatus for 10 min. The apparatus was cleaned thoroughly with 75% ethanol before each subject was tested. The movement of the mouse was video tracked and analyzed using ANY-maze software (USA). The open field was divided into central (center 20 cm diameter) and peripheral zones for analysis.

The elevated plus maze consisted of two open arms (35 cm long × 5 cm wide) and two closed arms of the same size with 10 cm high side walls. The apparatus was placed 50 cm above the ground. The tested mice were individually placed in the center and allowed for 10 min free exploration. The entries to and time spent in the open arms, center zone and closed arms were recorded. The maze was cleaned thoroughly with 75% ethanol between mice. The movement was tracked and analyzed using ANY-maze software (USA).

For three-chamber social interaction test, tree chambers (60 cm long × 40 cm wide × 22 cm high) connected by removable partitions in the plexiglass walls that allowed animals to freely move between the chambers. Mice were handled twice a day for 3 days before the test. Prior to the day of test, the handled mice were each habituated to the empty apparatus for 10 min. During the test, a stranger mouse was placed in a cylindrical wired cage (8 cm diameter, 17 cm high) with bars spaced 1 cm apart positioned in left and/or right chamber. The middle chamber was left empty all the time. Each test session had three stages: stage 1: 10 min habituation stage with two empty cages; stage 2: 5 min sociability test with a stranger mouse (S1) and an empty cage; stage 3: 5 min social memory test with the previously encountered stranger (S1) and a second novel stranger (S2). Each stage was separated by a 45 s-1 min interval. The amount of interaction was recorded using sniff time when the animal oriented its nose within 1 cm of the mouse contained in the wired cage. Sniff time was recorded by an experimenter using a stop-watch. Data were analyzed as a percentage time spent investigating the target cage over the total time interacting with either cage. The interaction was also tracked using ANY-maze software (IL, USA).

For five-trial social interaction test, the subject mouse was placed in a chamber (40 cm long × 20 cm wide × 22 cm high) and presented with a same sex juvenile, strange mouse in a cylindrical wired cage (8 cm diameter, 17 cm high) with bars spaced 1 cm apart. Six consecutive 1 min interaction trials with a 30–45 s inter-trial interval were used for each subject. On the last trial, a novel stranger juvenile mouse of the same sex was presented in the cage. A stop-watch was used to record the sniff time when the animal oriented its nose within 1 cm of the stranger mouse in the wired cage. The interaction was also tracked and analyzed using ANY-maze software (IL, USA).

### Statistical analyses

All the averaged data in the graphs were stated as mean ± SEM and statistically evaluated by Student’s t-test for comparisons of two groups or by ANOVA (one-way, two-way or repeated measures, as appropriate) for comparisons of more than two groups followed by post hoc Fisher’s LSD multiple comparison test using the SPSS program. P < 0.05 was considered significant. The details of statistical data, including statistical methods, P values and sample size, were provided in respective figure legends.

## Supplementary Information

**Additional file 1: Fig. S1**. Amyloid plaques in 6-month old APP/PS1 mice. Thioflavin-S staining images showing the presence of extensive amyloid plaques in 6-month old APP/PS1, but little staining signals in 3-month old APP/PS1 mice, particularly in the hippocampus. No amyloid plaques were found in 3-month or 6-month WT mice. Scale bar: 1000 μm.

## Data Availability

The data used in our study are available from the authors on reasonable request.

## Abbreviations

AD: Alzheimer’s Disease
AβO: Beta amyloid oligomers
LIMK1: Lim-domain protein kinase 1
APP/PS1: B6;C3-Tg(APPswe,PSEN1dE9)85Dbo/Mmjax
LTP: Long-term potentiation
TBS: Theta-burst stimulation
fEPSP: Field excitatory postsynaptic potential
