# Supplementary material for: Overexpression of LIMK1 in hippocampal excitatory neurons improves synaptic plasticity and social recognition memory in APP/PS1 mice
Source: Mol Brain. 2021 Jul 27;14:121. doi: 10.1186/s13041-021-00833-3 (PMC8314529; doi:10.1186/s13041-021-00833-3)
Supplement: Supplementary file 1 — Additional file 1: Fig. S1. Amyloid plaques in 6-month old APP/PS1 mice. Thioflavin-S staining images showing the presence of extensive amyloid plaques in 6-month old APP/PS1, but little staining signals in 3-month old APP/PS1 mice, particularly in the hippocampus. No amyloid plaques were found in 3-month or 6-month WT mice. Scale bar: 1000 μm. [file 13041_2021_833_MOESM1_ESM.pdf]

Additional file 1

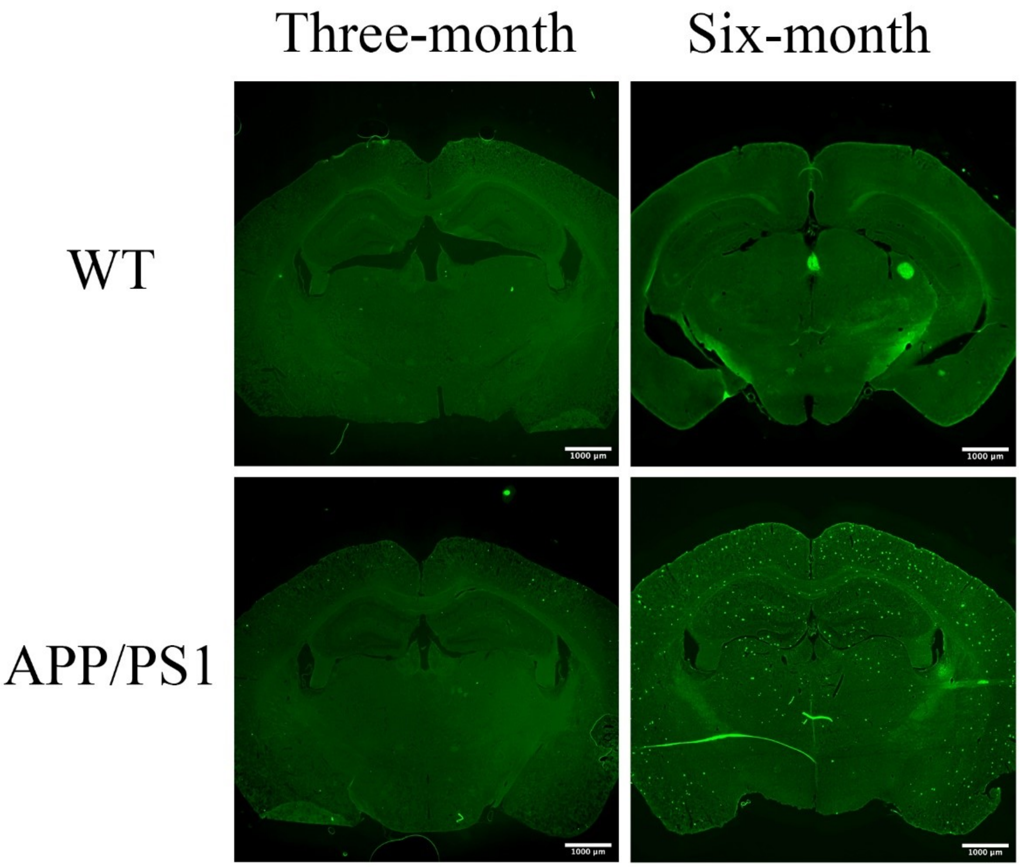

Figure S1. Amyloid plaques in 6-month old APP/PS1 mice. Thioflavin-S staining images showing the presence of extensive amyloid plaques in 6-month old APP/PS1, but little staining signals in 3-month old APP/PS1 mice, particularly in the hippocampus. No amyloid plaques were found in 3-month or 6-month WT mice. Scale bar: 1000 µm.
